# Resilience and social support as correlates of dementia risk: insights from a biopsychosocial analysis of the All of Us dataset

**DOI:** 10.3389/fpubh.2026.1772350

**Published:** 2026-08-26

**Authors:** Casey D. Xavier Hall, Zhuo Meng, Beth Okantey, Crim Sabuncu, Brittany Lane, Ladanya Ramirez Surmeier, Julia Sheffler, Diana C. Oviedo, Frank Y. Wong, Gabrielle B. Britton, Eugenia Millender

**Affiliations:** 1 Center of Population Sciences for Health Empowerment, College of Nursing, Florida State University, Tallahassee, FL, United States; 2 College of Social Work, Florida State University, Tallahassee, FL, United States; 3 Instituto de Investigaciones Científicas y Servicios de Alta Tecnología (INDICASAT AIP), Panamá, República de Panamá; 4 Department of Public Health, College of Social Sciences and Public Policy, Florida State University, Tallahassee, FL, United States; 5 Center for Translational Behavioral Sciences, Department of Behavioral Sciences and Social Medicine at the Florida State University College of Medicine, Tallahassee, FL, United States; 6 Escuela de Psicología, Universidad Santa María La Antigua, Panamá, República de Panamá; 7 Sistema Nacional de Investigación (SNI), Secretaría Nacional de Ciencia, Tecnología e Innovación (SENACYT), Panamá, República de Panamá

**Keywords:** biopsychosocial risk factors, CAIDE risk score, cognitive decline, dementia risk, resilience, social support

## Abstract

**Introduction:**

Cognitive decline is a growing concern in the United States as the population ages. Risk scores such as the Cardiovascular Risk Factors Aging and Incidence of Dementia (CAIDE) have been validated to assess long-term risk for cognitive decline. The existing literature has primarily focused on biological risk factors; however, there is a growing emphasis on integrating psychological and social factors that may contribute to a greater risk of cognitive decline.

**Methods:**

This study uses a large administrative data set (All of Us, *N* = 18,314) to examine biopsychosocial correlates of CAIDE risk score, examining psychological/behavioral correlates (e.g., anxiety, depression, sleep), social correlates (e.g., social support, loneliness), demographic correlates (e.g., sexual identity, race, gender), and biological correlates (e.g., pain). Correlates were examined using multiple linear regression with CAIDE score as the outcome, stratified by age group (<47 years, 47–53 years, >53 years).

**Results:**

Results varied by age strata. Among Age < 47 years, in comparison to heterosexuals, gay participants had increased CAIDE scores. Among Age < 47 and Age > 53, Both non-Hispanic Black and Hispanic participants had higher CAIDE scores than White participants. Across age groups, depression, stress, and resilience were associated with CAIDE. Social support was significant among Age < 45.

**Discussion:**

This analysis highlights the importance of framing risk for cognitive decline to incorporate both psychological and social determinants of health, in addition to biological risk factors.

## Introduction

One in five individuals in the U.S. will be 65 or older by 2030 (1) and by 2034, the aging population is set to outnumber those under 18 for the first time in history (2). The rapidly aging U.S. population highlights the need for an understanding of age-related health concerns such as cognitive impairment and dementia, as well as related factors that contribute to cognitive decline throughout the life course. One study utilizing nationally representative data found mild cognitive impairment occurs in 22% of U.S. adults 65 and older, and dementia occurs in 10% of U.S. adults 65 and older, which increases to 35% among those 90 and older (3). While overall, the incidence of dementia in the U.S. is decreasing, individuals living with dementia are increasing due to the rising aging population (4). Much of the dementia literature has focused on biological indicators and processes, such as cardiovascular indices, and behavioral interventions targeting these processes, such as diet and exercise (5, 6). Despite a growing understanding of psychosocial correlates of cardiovascular disease (7), psychological and social determinants of dementia remain underexamined (8).

The Cardiovascular Risk Factors, Aging and Dementia Study (CAIDE) Dementia Risk Score was developed and standardized in a Finnish population to predict the risk of dementia in middle-aged individuals 20 years later, based on risk factors including age, sex at birth, education, body mass index, systolic blood pressure, total cholesterol, and physical activity (9). The score has been highly predictive of cognitive decline in prospective cohorts (10, 11).

Dementia researchers have begun to emphasize psychological and social determinants of health as relevant mechanisms impacting cognitive decline and the biological processes typically identified as risk factors (e.g., cardiovascular disease). These examinations have included a range of factors including community and social contexts (e.g., social isolation, rurality, discrimination, neighborhood deprivation), socio-economic factors (e.g., socio-economic status, employment, educational attainment), structural factors (e.g., healthcare access), psychological factors (e.g., depression, anxiety, stress) as well as demographic factors (e.g., ethnic minority status), which are often associated with patterning of psychological or social determinants (12–21). However, social determinants of health frameworks are relatively new to research addressing cognitive health outcomes (20), despite the emphasis on SDOH in cardiovascular health literature (22).

## Current study

**Figure 1:**
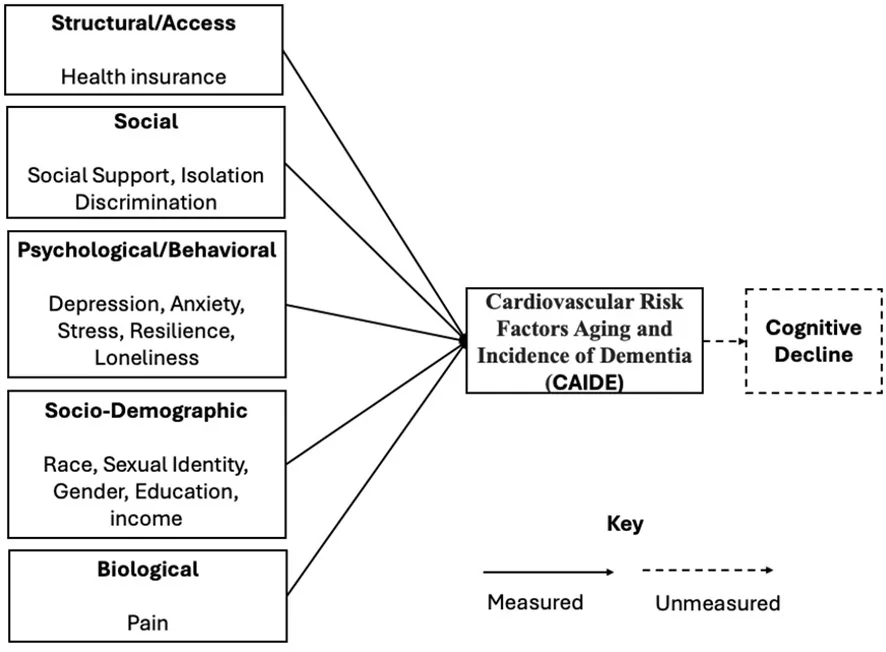
Conceptual framework of biopsychosocial determinants of CAIDE.

Given the aforementioned gaps in the literature, the present analyses seeks to examine biopsychosocial correlates of CAIDE scores among adults in a large administrative dataset (All of Us- AoU) stratified by age groups (See Figure 1; < 47 years, 47–53 years, >53 years). Aligned with our Conceptual Framework (see Figure 1), we include variables conceptualized as biological, psychological, and social. We hypothesize that a mix of biological, psychological, and social factors will be significantly associated with CAIDE score outcomes.

## Materials and methods

### Participants

We conducted this study using data from AoU (clinical trial number: not applicable), an NIH-funded program aimed at accelerating health research and improving health care through data collected from millions of people living in the United States (23). Participants enrolled in AoU would have basic information, such as their demographics and socioeconomics, collected through The Basics survey. If the participants consented to their electronic health records (EHR), that information would also be collected, and they might be invited to a free in-person appointment for some physical measurements such as height, weight, blood pressure, and/or blood or saliva samples. Participants could consent to receive their DNA results. They might complete more surveys and/or connect a wearable device such as a Fitbit tracker or a smartwatch to participate in the program further. Controlled Tier Dataset v7, comprising 413,360 participants enrolled from May 2017 to July 2022. As of July 1, 2022, the total number of participants in the retrieved dataset was 413,360. Considering the CAIDE score and most of the correlates of interest were available after 2020, participants with an available CAIDE score from 2020 onward were eligible for this study. Participants diagnosed with dementia before their available CAIDE score were excluded, resulting in a total sample size of *N* = 18,314 participants.

### Measures

#### Demographics

Age, gender identity (GI), sexual orientation (SO), race/ethnicity, education, annual household income, and health insurance were included in the study. Age was calculated using the field “date of birth” from the prepackaged concept “Demographics” in AoU. Gender identity (GI) contained categories of cisgender man, cisgender woman, non-binary, transgender, and other. Sexual orientation (SO) contained categories of heterosexual, homosexual, bi+ (encompassing bisexual, pansexual and other identities with multi-gender attraction), and other identity. Detailed components of each GI and SO category were summarized in Supplementary Table S1. Race/ethnicity consisted of seven categories: non-Hispanic white, non-Hispanic black, non-Hispanic Asian & Native Hawaiian and Pacific Islander (NHPI), non-Hispanic other race, Hispanic only, and multi-race/ethnicity, and another race/ethnicity. Education ranged from never attended to an advanced degree and was categorized into three groups: less than a college degree, college graduates, and individuals with advanced degrees. Annual household income was categorized into four levels by cutoffs of $35,000, $75,000, and $150,000. Health insurance was grouped into private, public, both, and other/none, where private included participants whose healthcare plans were self-purchased and/or from their employers, and public included participants who had Medicare, Medicaid, military health care, Veteran Affairs (VA) health care, and/or Indian Health Service.

#### Predictors

Pain was obtained from the Overall Health survey and EHR domain Labs and Measurements. In the Overall Health survey, pain was measured by a single question asking participants to rate their average pain in the past 7 days from 0 to 10, where 0 represented no pain, and 10 represented the worst pain imaginable. In Labs and Measurements, pain value was obtained from three unique concepts (see Supplementary Table S2). Most of the values fell into the range of 0 to 10. Values smaller than 0 or greater than 10 were excluded as potential outliers or errors.

Depression was obtained from the survey COVID-19 Participant Experience (COPE), EHR domains, Lab and Measurements, and Observations in AoU. The participants completed multiple versions of the COPE survey. The nine-item Patient Health Questionnaire (PHQ-9) was adopted in the May 2020 – July 2020 versions, while the first two items of the questionnaire (PHQ-2) were assessed in the Nov. 2020 – Feb. 2021 versions (24, 25). Participants were asked to rate how often they experienced depressive symptoms in the past two weeks on a four-point Likert-type scale from 0 = “Not at all” to 3 = “Nearly every day”. A sum ranging from 0 to 27 for PHQ-9 or 0 to 6 for PHQ-2 was calculated, where a higher score indicated a higher level of depression. Some participants had their PHQ-9 and/or PHQ-2 sum scores available in their EHR domains (see Supplementary Table S2). An average score was calculated if a participant had multiple observations within a year. The sum scores were standardized with respect to different scales.

Anxiety was obtained from the COPE survey and EHR Observations. The 7-item and 2-item Generalized Anxiety Disorder Scale (GAD-7 and GAD-2) were utilized in the May 2020 – July 2020 versions and the Nov. 2020 – Feb. 2021 versions of the COPE survey (26, 27). Participants reported the frequency of anxiety they experienced in the past two weeks on a four-point scale ranging from 0 = “Not at all” to 3 = “Nearly every day”. A sum score was calculated for both GAD-7 (range: 0–21) and GAD-2 (range: 0–6), with higher scores indicating greater severity of generalized anxiety symptoms. A GAD-7 sum score was available for some participants in their EHR under Observations. All sum scores were standardized according to their respective scales.

Stress was obtained from the surveys Social Determinants of Health (SDOH) and COPE. Both surveys used the 10-item Cohen's Perceived Stress Scale (PSS) to evaluate how often participants felt or thought specific ways on a five-point Likert-type scale, where 0 represented “Never” and 4 represented “Very often” (28). Note that items 4, 5, 7, and 8 described positive feelings and thoughts, while the others described negative feelings. The positive items were reverse-coded so that a higher sum score (range: 0–40) indicated a higher level of perceived stress.

The Everyday Discrimination Scale measured discrimination in the surveys SDOH and COPE (29). Participants were asked to report the frequency with which they experienced nine different forms of discrimination. All six recommended response categories were adopted in the survey SDOH, while only four categories were used in COPE. Values of 1 to 6 were assigned to the six categories, ranging from “Never” to “Almost every day,” to ensure consistency in the sum scores between surveys. Higher sum scores (range: 9–54) suggested more frequent discrimination experiences.

Loneliness was measured using the 8-item short form of the revised UCLA Loneliness Scale (ULS-8) in the surveys SDOH and COPE (30). Participants were asked to rank how often they felt lonely in different perspectives from 1 = “Never” to 4 = “Often.” Two items (items 3 and 6) with positive wording were reverse-coded. The sum score, ranging from 8 to 32, was calculated to indicate a participant's level of loneliness.

Social support was assessed by the RAND MOS Social Support Survey Instrument in the SDOH and COPE surveys (31). Eight everyday items from the Instrument (19 items in total) were included in both surveys. These items were rated on a scale from 1 = “None of the time” to 5 = “All of the time”, reflecting the different types of social support participants received. An average score was calculated (range: 1–5), with higher values representing better social support.

Resilience and coping were assessed using the four-item Brief Resilient Coping Scale (BRCS) in the COPE survey. Participants rated the extent to which they agreed that the items reflected their positive responses to difficulties and stress (32). Each item was rated on a five-point Likert-type scale ranging from 1 = “Does not describe me at all” to 5 = “Describes me very well.” A sum score that ranged from 4 to 20 was calculated, such that higher scores represented better resilience and coping skills.

#### Outcome

Dementia risk was measured by the CAIDE score, which was the outcome of interest (9). The CAIDE score was developed in a middle-aged cohort using risk factors including age, sex at birth, education, systolic blood pressure, body mass index, total cholesterol, and physical activity to predict their risk of developing dementia in 20 years. Data for these seven risk factors were extracted from surveys, Fitbit data, and EHR, then calculated and/or categorized for CAIDE score calculation (see Supplementary Tables S2 and S3 for details). A total score of 0 – 15 was calculated to represent dementia risk. The CAIDE score was also dichotomized using a cutoff of 6 for sensitivity analyses, consistent with prior research (33).

### Statistical analysis

The analyses were conducted using R through the AoU workbench. For each correlate and risk factor from each data source, a yearly average was calculated if a variable was measured multiple times within the same year. A hierarchical source selection approach was applied, where each variable was drawn from a single best available source according to a predefined ranking (see Supplementary Table S2). Survey-based measures were generally prioritized over EHR-derived measures; however, sources providing continuous measured values were prioritized over those reporting only binary or categorical condition classifications. Within EHR sources, records with specific concept names were prioritized over ambiguous ones to minimize misclassification. For depression and anxiety, where two validated instruments were used (PHQ-9/PHQ-2 and GAD-7/GAD-2 respectively), scores were standardized within each instrument prior to analysis. Variables retrieved from their top available data source were merged by participant ID and year to form our dataset. Demographics collected from The Basics survey were merged into the dataset by participant ID, as they were only collected once. Participants with CAIDE scores since 2020 who had not been diagnosed with dementia were retained in the study, resulting in a baseline sample size of 18,314.

Descriptive statistics were used to describe sample characteristics. A hierarchical regression scheme was applied to explore associations between dementia risk and the correlates. First, the effects of demographics on CAIDE scores were analyzed by linear regression. Considering that age and education were accounted for in CAIDE score calculation and gender identity had a similar distribution to sex at birth, only sexual orientation, race/ethnicity, income, and health insurance were brought into further analyses as control variables. Heterosexual, non-Hispanic white, less than $35,000, and private insurance served as their corresponding reference groups. Participants who preferred not to answer or skipped the questions were grouped into the “Missing” category.

Next, the bivariate linear association between each correlate and CAIDE score was evaluated after adjusting for demographics, with Benjamini–Hochberg false discovery rate correction applied to address multiple comparisons. Note that the bivariate analyses and the final model were stratified by age using the cutoffs defined by the CAIDE scoring algorithm (< 47, 47–53, >53 years) due to its impact on the risk factors. Similar analyses for the dichotomized CAIDE dementia risk using logistic regression were conducted as sensitivity analyses due to the value of predicting cognitive risk and to ensure consistency in the conclusions. To assess the missing data mechanism, the likelihood of having any missing data was modeled as a function of demographic characteristics using logistic regression, which indicated a missing at random (MAR) mechanism. Listwise deletion was used as the primary analytic approach, with multiple imputation subsequently conducted as a sensitivity analysis using the mice package in R (*m* = 5, predictive mean matching for continuous variables), with all analytic variables including the outcome included in the imputation model. Imputation-based results are reported in the Supplementary materials. As only 8% of participants had valid pain data, pain was examined as a standalone measure and was excluded from FDR correction and multivariate analyses to avoid introducing substantial missingness into the final model.

## Results

### Sample characteristics

The overall median CAIDE score was 6 (Mean = 5.6, SD = 2.6), indicating a moderately high risk of dementia. Sample characteristics are summarized in Table 1. The average age of these participants was 59.3 years (SD = 14.7). About 70% were older than 53; about 20% and 10% were younger than 47 years and in between, respectively. Regarding gender and sexuality, most participants were cisgender (98.8%) and heterosexual (91.0%). Approximately one-third of the participants were cisgender men, while the number of cisgender women was about twice that. Less than 1% of them were gender minorities. About 3.8%, 3.5%, and 0.6% were homosexual, bi-sexual, and with another SO. Participants who self-identified as bi+ or with another SO were associated with lower dementia risk compared to heterosexual participants. Among these participants, 80.8% were non-Hispanic white. The next two most frequent race/ethnicity groups were non-Hispanic black (6.7%) and Hispanic only (4.6%). Compared to non-Hispanic whites, non-Hispanic black participants had a higher risk of developing dementia. In contrast, participants who were non-Hispanic Asian & NHPI, Hispanic, or with multi-race/ethnicity were associated with a lower risk. The education level of this sample was relatively high, with a similar number of participants (about 32%) not holding and holding a college degree, and slightly more participants (36.9%) having advanced degrees. Most of their annual household income fell into the $75,000–$149,999 category. Increased income led to decreases in the CAIDE score. Most participants (43.8%) had private insurance, while 27.2% had public insurance. About 17.8% had both, and 1.9% had other healthcare plans or no coverage. Compared to participants with private health insurance, participants who belonged to the other categories were at higher risk for dementia.

**Table 1:** Characteristics of Adult Sample from the All of Us Dataset (*n* = 18,314).

		CAIDE score		
Demographics	Count (%)	Mean (SD)	β (SE)	*p*-value
Age^*^				
Less than 47 years	3,790 (20.7%)	2.202 (1.792)	ref	ref
47–53 years	1,864 (10.2%)	5.612 (1.914)	3.411 (0.053)	< 0.001
Greater than 53 years	12,660 (69.1%)	6.683 (1.910)	4.482 (0.035)	< 0.0 001
Gender Identity^*^				
Cisgender man	6,191 (33.8%)	6.561 (2.342)	ref	ref
Cisgender woman	11,907 (65.0%)	5.187 (2.594)	−1.374 (0.039)	< 0.001
Non-binary	82 (0.4%)	3.451 (2.846)	−3.110 (0.279)	< 0.001
Transgender	42 (0.2%)	4.738 (3.069)	−1.823 (0.389)	< 0.001
Another Identity	14 (0.1%)	4.286 (3.024)	−2.275 (0.672)	0.001
Missing	78 (0.4%)	6.346 (2.203)	−0.215 (0.286)	0.453
Sexual orientation				
Heterosexual	16,668 (91.0%)	5.706 (2.568)	ref	ref
Gay/lesbian	694 (3.8%)	5.759 (2.565)	0.053 (0.100)	0.594
Bi+	650 (3.5%)	4.057 (2.779)	−1.649 (0.103)	< 0.001
Another identity	117 (0.6%)	4.299 (3.174)	−1.407 (0.239)	< 0.001
Missing	185 (1.0%)	6.319 (2.463)	0.613 (0.191)	0.001
Race/ethnicity				
Non-Hispanic White	14,794 (80.8%)	5.680 (2.555)	ref	ref
Non-Hispanic Black	1,222 (6.7%)	6.290 (2.563)	0.610 (0.077)	< 0.001
Non-Hispanic Asian & NHPI	451 (2.5%)	4.244 (2.602)	−1.436 (0.123)	< 0.001
Non-Hispanic Other	232 (1.3%)	5.526 (2.719)	−0.154 (0.171)	0.366
Hispanic	834 (4.6%)	5.297 (2.792)	−0.383 (0.092)	< 0.001
Multi-race/ethnicity	529 (2.9%)	4.660 (2.894)	−1.020 (0.114)	< 0.001
Missing	260 (1.4%)	6.433 (2.312)	0.753 (0.164)	< 0.001
Education^*^				
Less than a college degree	5,822 (31.8%)	6.486 (2.618)	ref	ref
College graduate	5,727 (31.3%)	5.263 (2.571)	−1.222 (0.047)	< 0.001
Advanced degree	6,765 (36.9%)	5.249 (2.432)	−1.237 (0.045)	< 0.001
Income				
Less than $35,000	2,936 (16.0%)	6.268 (2.799)	ref	ref
$35,000 – $74,999	4,344 (23.7%)	5.768 (2.682)	−0.500 (0.061)	< 0.001
$75,000 – $149,999	5,807 (31.7%)	5.503 (2.520)	−0.766 (0.058)	< 0.001
$150,000 and more	3,645 (19.9%)	5.088 (2.403)	−1.180 (0.064)	< 0.001
Missing	1,582 (8.6%)	5.976 (2.383)	−0.292 (0.080)	< 0.001
Health insurance				
Private	8,030 (43.8%)	4.792 (2.703)	ref	ref
Public	4,978 (27.2%)	6.487 (2.335)	1.694 (0.045)	< 0.001
Both	3,263 (17.8%)	6.529 (1.974)	1.737 (0.051)	< 0.001
Other/None	356 (1.9%)	5.531 (2.836)	0.739 (0.134)	< 0.001
Missing	1,687 (9.5%)	5.554 (2.489)	0.762 (0.066)	< 0.001

^*^ These measures are not used for further regression analyses.

### Regression analyses

The effect of each correlate on the CAIDE dementia risk score was evaluated by linear regression, adjusted for sexual orientation, race/ethnicity, income, and health insurance. Surprisingly, several correlates, such as depression, anxiety, perceived stress, discrimination, and loneliness, showed significant associations with the CAIDE score, but in the opposite direction to the hypotheses. This motivated us to explore further the relationships between these correlates and the CAIDE risk factors, considering that they may help explain the reversed effects. Age, as one of the CAIDE risk factors, was correlated with all these risk factors the most. Specifically, older age was linked to better mental health and less discrimination and loneliness. Therefore, we stratified the analyses by age based on age groups used for CAIDE score calculation.

The results of the bivariate analyses are presented in Table 2. Serving as a standalone measure with limited data, more severe pain was associated with higher dementia risk across all age groups. Among participants younger than 47 years, all correlates were significantly associated with CAIDE risk. Participants who experienced worse depression, higher anxiety and stress, more frequent discrimination, and greater loneliness tended to have an increased risk of dementia. Enhanced social support and improved resilience and coping skills could lower the dementia risk. For participants aged between 47 and 53 years, worse mental health was associated with higher dementia risk, while resilience was associated with lower dementia risk. Notably, the associations between depression (β = 0.208, SE = 0.042, *p* < 0.001, *q* < 0.001), resilience (β = −0.224, SE = 0.048, *p* < 0.001, *q* < 0.001), and the CAIDE score remained significant following both multiple imputation and FDR correction. Within participants older than 53 years, a higher level of depression (β = 0.207, SE = 0.020, *p* < 0.001, *q* < 0.001), stress (β = 0.071, SE = 0.019, *p* < 0.001, *q* < 0.001), and discrimination experience (β = 0.131, SE = 0.021, *p* < 0.001, *q* < 0.001) resulted in a higher risk of developing dementia. Both social support and resilience and coping appeared to help lower the dementia risk, though not as much as in the younger cohort. Findings in this age group were largely consistent across listwise deletion and multiple imputation, with multiple imputation yielding more stable results following FDR correction.

**Table 2:** Linear regression results for each correlate of CAIDE score adjusted for demographics and stratified by age group.

		CAIDE score		
Predictor	*N* (%)	β (SE)	*p*-value	FDR-corrected *q*
Age < 47 years, *N* = 3,790				
Pain	332 (8.8%)	0.166 (0.101)	0.102	NA
Depression	3,045 (80.3%)	0.227 (0.027)	< 0.001	< 0.001
Anxiety	3,017 (79.6%)	0.107 (0.027)	< 0.001	< 0.001
Stress	3,215 (84.8%)	0.149 (0.032)	< 0.001	< 0.001
Discrimination	3,213 (84.8%)	0.202 (0.026)	< 0.001	< 0.001
Loneliness	3,220 (85.0%)	0.155 (0.031)	< 0.001	< 0.001
Social support	3,215 (84.8%)	−0.224 (0.033)	< 0.001	< 0.001
Resilience	2,853 (75.3%)	−0.250 (0.032)	< 0.001	< 0.001
Age 47–53 years, *N* = 1,864				
Pain	194 (10.4%)	0.331 (0.139)	0.018	NA
Depression	1,559 (83.6%)	0.212 (0.043)	< 0.001	< 0.001
Anxiety	1,548 (83.0%)	0.105 (0.044)	0.016	0.028
Stress	1,625 (87.2%)	0.101 (0.047)	0.033	0.046
Discrimination	1,621 (87.0%)	0.098 (0.040)	0.015	0.028
Loneliness	1,629 (87.4%)	0.040 (0.046)	0.378	0.378
Social support	1,629 (87.4%)	−0.070 (0.049)	0.153	0.179
Resilience	1,488 (79.8%)	−0.245 (0.047)	< 0.001	< 0.001
Age > 53 years, *N* = 12,660				
Pain	1,648 (13.0%)	0.228 (0.045)	< 0.001	NA
Depression	11,350 (89.7%)	0.207 (0.020)	< 0.001	< 0.001
Anxiety	11,298 (89.2%)	0.029 (0.021)	0.160	0.187
Stress	11,594 (91.6%)	0.071 (0.019)	< 0.001	< 0.001
Discrimination	11,578 (91.5%)	0.131 (0.021)	< 0.001	< 0.001
Loneliness	11,625 (91.8%)	0.024 (0.018)	0.190	0.190
Social support	11,601 (91.6%)	−0.036 (0.018)	0.046	0.064
Resilience	10,960 (86.6%)	−0.175 (0.018)	< 0.001	< 0.001

All predictors were standardized.

Significant mental health and social determinants of health correlates identified in the bivariate analyses were brought into the final models, along with demographics. The results are presented in Table 3. For participants younger than 47 years old, significant associations were observed for all key correlates. However, anxiety, stress, and loneliness showed associations in the opposite direction to hypotheses. VIF values slightly above three suggested mild collinearity among depression, anxiety, and perceived stress, which may partly explain the reversed directions. In sensitivity analyses fitting each variable separately alongside other covariates, the association between depression and the CAIDE score remained significant, while those for anxiety and perceived stress became nonsignificant, suggesting that their associations in the full model may be influenced by multicollinearity (see Supplementary Table S4). Among participants aged 47 – 53 years, depression (β = 0.409, SE = 0.072, *p* < 0.001) and resilience (β = −0.243, SE = 0.049, *p* < 0.001) remained significant in the multivariate analysis, consistent with the bivariate findings. Stress exhibited a negative association in the full model that became nonsignificant when fitted separately, suggesting potential multicollinearity influence. Findings among participants older than 53 years were consistent in terms of the direction and significance of associations.

**Table 3:** Adjusted linear regression results with CAIDE outcomes stratified by age group.

	CAIDE score								
	Age < 47 years (*N* = 2,836)			Age 47–53 years (*N* = 1,467)			Age > 53 years (*N* = 10,826)		
Measures	β (SE)	*p*-value	VIF	β (SE)	*p*-value	VIF	β (SE)	*p*-value	VIF
*Predictors*									
Depression	0.375 (0.050)	< 0.001	3.6	0.409 (0.072)	< 0.001	3.0	0.316 (0.031)	< 0.001	2.3
Anxiety	−0.165 (0.048)	0.001	3.3	−0.103 (0.078)	0.186	3.3	-	-	-
Stress	−0.134 (0.060)	0.026	3.6	−0.270 (0.085)	0.001	3.3	−0.248 (0.030)	< 0.001	2.5
Discrimination	0.152 (0.031)	< 0.001	1.4	0.068 (0.045)	0.129	1.3	0.095 (0.023)	< 0.001	1.2
Loneliness	−0.156 (0.048)	0.001	2.5	-	-	-	-	-	-
Social support	−0.156 (0.042)	< 0.001	1.7	-	-	-	-	-	-
Resilience	−0.216 (0.035)	< 0.001	1.2	−0.243 (0.049)	< 0.001	1.2	−0.163 (0.019)	< 0.001	1.2
*Demographics*									
Sexual orientation									
Heterosexual	Ref	Ref		Ref	ref		ref	ref	
Gay/Lesbian	0.393 (0.149)	0.008		0.368 (0.208)	0.078		0.274 (0.092)	0.003	
Bi+	−0.013 (0.113)	0.907		0.264 (0.245)	0.282		0.117 (0.138)	0.397	
Another identity	−0.132 (0.249)	0.597		−0.686 (0.596)	0.250		0.352 (0.327)	0.282	
Missing	−0.097 (0.368)	0.793		−0.214 (0.531)	0.687		0.170 (0.175)	0.330	
Race/ethnicity									
Non-Hispanic White	Ref	Ref		Ref	ref		ref	ref	
Non-Hispanic Black	0.516 (0.129)	< 0.001		0.506 (0.147)	0.001		0.567 (0.076)	< 0.001	
Non-Hispanic Asian&NHPI	−0.361 (0.150)	0.016		−0.509 (0.246)	0.039		−0.325 (0.136)	0.017	
Non-Hispanic Other	−0.153 (0.263)	0.561		−0.918 (0.632)	0.146		−0.041 (0.272)	0.879	
Hispanic	0.650 (0.113)	< 0.001		0.160 (0.150)	0.286		0.319 (0.094)	0.001	
Multi-race/ethnicity	0.133 (0.132)	0.312		0.151 (0.314)	0.631		0.139 (0.202)	0.492	
Missing	−0.230 (0.517)	0.656		−0.368 (0.506)	0.468		0.123 (0.129)	0.340	
Income									
Less than $35,000	Ref	Ref		Ref	ref		ref	ref	
$35,000 – $74,999	−0.240 (0.104)	0.021		−0.578 (0.157)	< 0.001		−0.337 (0.059)	< 0.001	
$75,000 – $149,999	−0.221 (0.108)	0.041		−0.727 (0.156)	< 0.001		−0.461 (0.058)	< 0.001	
$150,000 and more	−0.464 (0.120)	< 0.001		−1.127 (0.162)	< 0.001		−0.764 (0.065)	< 0.001	
Missing	−0.154 (0.149)	0.301		−0.384 (0.198)	0.053		−0.507 (0.073)	< 0.001	
Health insurance									
Private	Ref	Ref		Ref	ref		ref	ref	
Public	0.706 (0.104)	< 0.001		0.129 (0.150)	0.390		0.270 (0.045)	< 0.001	
Both	0.457 (0.193)	0.018		0.120 (0.259)	0.644		0.177 (0.046)	< 0.001	
Other/None	0.214 (0.205)	0.297		−0.072 (0.327)	0.826		0.127 (0.134)	0.342	
Missing	0.133 (0.108)	0.218		0.009 (0.148)	0.505		−0.094 (0.061)	0.127	

All continuous predictors were standardized.

### Sensitivity analyses

The bivariate associations between each correlate and the dichotomized CAIDE dementia risk, adjusted for demographics, are presented in Supplementary Table S5. Higher depression, stress, discrimination, and lower resilience were associated with higher dementia risk across all age groups. Additionally, social support was associated with lower dementia risk among participants younger than 47 years, and anxiety was linked to higher dementia risk among participants aged 47–53 years.

In the final models, including significant correlates together concerning age groups, only discrimination remained significant in explaining the CAIDE dementia risk for participants with relatively younger ages (< 47 years). The conclusions of the other two age groups were mainly consistent with previous analyses. Specifically, higher depression would increase dementia risk, while better resilience would reduce it for participants aged 47 to 53 years. Discrimination additionally accounted for dementia risk in the relatively older age group. However, anxiety and stress showed associations in unexpectedly directions, which became nonsignificant when each was fitted separately, suggesting potential multicollinearity influence. The results of the final logistic models are shown in Supplementary Table S6. Results based on multiple imputation are presented in Supplementary Tables S7–S10. Findings were largely consistent with the listwise deletion analyses, with minor differences in the significance of a few correlates as noted above.

## Discussion

These analyses emphasize the importance of a biopsychosocial understanding of correlates with cognitive risk across the lifespan. CAIDE and other similar probabilistic risk scores predominantly comprise cardiovascular and behavioral indicators such as exercise and blood pressure (5). However, we know that biopsychosocial correlates such as SDOH impact cardiovascular health (7) and, therefore, likely impact cognitive risk scores like CAIDE. Thus, there is a critical need to consider psychosocial correlates of risk for dementia (8), apart from biological correlates and physical health factors (34).

The present analysis identified demographic, psychological and behavioral, social, and biological correlates with CAIDE, suggesting multi-level and multi-faceted factors contributing to probabilistic risk for cognitive decline and attenuated risk. First, we identified several demographic factors correlated with increased CAIDE scores, including Black and Hispanic/Latino identities. Asian participants had a lower risk compared to the White population. These differences by race align with previous literature, which has identified Black and Hispanic/Latino populations as having a higher risk for cardiovascular disease (35), hypertension, a significant risk factor for cardiovascular disease (36), and cognitive impairment (37, 38). We also observed elevated CAIDE scores among people reporting bi+ identities and heterosexuals in bivariate analyses and between gay identities relative to heterosexuals in multivariate analyses. Though it should be interpreted with caution given small cell sizes, this seems to be a novel finding as the authors could not identify previous peer-reviewed comparisons between gay and heterosexual participants' CAIDE scores. This novel finding suggests the need for more in-depth exploratory work with sexual minorities, as the authors are not aware of previous work comparing CAIDE across sexual or gender identities. It is important to note that demographic differences based on socially constructed categories such as race or sexual identity are likely proxies for underlying social processes. These socially constructed categories are not risk factors in themselves. One possible pathway that could be explored in the future is the Minority Stress Framework, which is one of the most prevailing theories in sexual and gender minority health disparities (39), though it has not been thoroughly examined in relation to CAIDE and dementia. Some possible factors, including SDOH, such as discrimination and socioeconomic status, may drive racial differences (40, 41). For instance, the fear of stigma may be a reason that individuals delay or avoid seeking medical attention (42). Our present analysis did measure discrimination and access to insurance finding associations with CAIDE Scores; however, minoritized demographics remain associated with CAIDE outcomes suggesting that additional factors may be at play. Other possible related factors that remain unexamined in the present analysis are for instance, differential access to healthcare by identity or cultural differences in food or exercise. More research is needed to examine what additional pathways might be driving these demographic differences.

Second, regarding psychological and behavioral factors, we identified depression as a correlate across age groups. This is backed by previous literature (43), and some risk scores incorporate depression in their calculation (44). The more novel finding in this area is that this analysis suggests resilient coping was negatively associated with CAIDE scores across age groups, suggesting it may warrant further investigation as a potential target for dementia risk reduction. Resilience and coping skills interventions based in Cognitive-Behavioral Therapy, emotion regulation skills and mindfulness techniques, have been shown to be effective in increasing coping skills (45, 46). These interventions have also been adapted for specific groups such as sexual minority men and racial minority groups (47, 48). Further longitudinal research should examine whether existing resilience and coping skills interventions (49) may contribute to reducing probabilistic dementia risk, and exploratory research should consider their adaptation for diverse populations.

Third, regarding SDOH, two factors were identified. Discrimination was associated with an increased CAIDE score. While previous work has linked discrimination to both cardiovascular indicators (50) and cognitive decline (40, 41), we are not aware of previous research showing an association with CAIDE. This finding supports the need for greater integration of SDOH in dementia research and may have implications for demographic disparities in cognitive decline and intervention (8). Future research may attempt to clarify discrimination, types, sources, or settings that have the most significant impact on CAIDE to assist in the scoping of intervention, for instance, whether the associated discrimination occurs in a specific setting, such as a medical setting.

Social support was identified as a SDOH correlate with reduced CAIDE score. Existing research does identify social support as a protective factor and social isolation as a risk factor for cognitive decline (51). While there might be a number of explanations for the relationship between social support and dementia, one explanation is that increased social support connotes more social activities or interactions, which lead to increased cognitive stimulation (52, 53). The association between social support and CAIDE in our findings further supports that the impact of social support may be upstream and a contributor to risk factors for cognitive decline. Indeed, previous research on social support interventions such as support groups suggests some improved outcomes such as quality of life among older adults with dementia (54) and mixed findings on frailty outcomes (55). However, examination of such interventions in relation to long term probabilistic dementia risk is limited. Exploratory research should consider whether social support interventions have potential as reduction strategies for probabilistic risk in midlife prior to signs of cognitive decline.

Lastly, this analysis attempted to examine pain as a biological correlate, with significant correlations with CAIDE in exploratory bivariate analyses; however, due to low availability of pain data in this sample we were unable to fully assess this correlate. Though recent meta-analyses suggested that pain is not associated with cognitive decline itself (56), it has been suggested that pain may be associated with the CAIDE score through reduced physical exercise (57). Further research is needed to consider pain in its relation to CAIDE and dementia risk. We frame pain as a biological factor; however, in this analysis, pain is measured as subjective pain and not through objective measurement. It may be important to consider the differences between subjective and objective measurements of main and their association with CAIDE as this may have implications for intervention.

While this study has strengths, such as the use of a large administrative dataset, these results should be understood considering limitations. First, this analysis is cross-sectional, which limits our ability to observe the directionality of relationships. The parent study used non-probability sampling, which limits the generalizability of these findings. Future studies should consider representative sampling and longitudinal techniques to better characterize these associations. Second, as the CAIDE score represents a probabilistic risk estimate rather than a clinical diagnosis of dementia or a direct measure of cognitive decline, findings should be interpreted in the context of dementia risk as operationalized by this instrument. Additionally, as age and education are embedded components of the CAIDE score, the observed associations should be interpreted as primarily reflecting the relationship between the examined correlates and the modifiable components of dementia risk, given that age was held constant through stratification and education showed minimal variability in our sample (92% above high school per CAIDE criteria). While this stratification approach improves interpretability within each stratum, comparisons of regression estimates across strata should be made with caution, as older strata begin at a structurally higher CAIDE baseline and stratum-specific estimates may not be directly comparable in magnitude. Furthermore, the negative associations between anxiety, stress, and CAIDE scores observed in the full model were counterintuitive and inconsistent with existing literature. However, the precise mechanism underlying these unexpected associations remains unclear. Sensitivity analyses in which each variable was fitted separately revealed that these associations became nonsignificant, while depression remained significant and in the expected direction. This pattern suggests that the unexpected associations may be partially attributable to multicollinearity among psychosocial correlates, though other explanations cannot be ruled out, including suppression effects among correlated psychosocial correlates, potential survivorship bias given that individuals with poorer mental health may have been excluded due to prior dementia diagnosis, or the possibility that the true relationships between these constructs and dementia risk are indeed negative in this population. Future studies should consider alternative modeling strategies, such as composite psychosocial measures or regularization methods, to better disentangle the independent contributions of correlated psychosocial constructs to dementia risk. The effect sizes in our analysis range between 0.1 and0.4. A CAIDE score of 0–5 translates to approximately 1% risk of dementia and this percentage increases with each incremental range: 6–7 to 1.9%, 8–9 to 4.2%, 10–11 to 7.4%, and 12–15 to 16.4% (58). While an increase of 0.1–0.4 may be a small increase in CAIDE score on its own. While a single unit increase in a continuous correlate like a depression score is unlikely to be associated with a change in overall risk category, higher scores in correlate scales and in combination with other correlates may represent a change in risk category as assessed by the CAIDE score. Demographic findings should be considered within the context of small relative cell sizes as the parent study was not designed to capture minority populations. However, these preliminary signals may be examined in studies that are powered to examine these comparisons across populations. Lastly, a substantial portion of the psychosocial data was collected during the COVID-19 pandemic, which may affect the generalizability of findings to non-pandemic periods.

This analysis demonstrates a need to further integrate social and psychological factors into research examining the risk for cognitive impairment. In particular, newer areas for further research include gay/lesbian populations and resilience/coping as possible protective factors for dementia risk. These findings also suggest several avenues of potential intervention to reduce risk for cognitive impairment, including coping skills, social support, mindfulness skills, and cognitive behavioral therapy.

## Data Availability

Publicly available datasets were analyzed in this study. This data can be found here: https://www.researchallofus.org (National Institutes of Health All of Us dataset). However, restrictions apply, and availability is contingent upon registration and institutional access.
